# Natural Deep Eutectic Solvent Extraction of Flavonoids of *Scutellaria baicalensis* as a Replacement for Conventional Organic Solvents

**DOI:** 10.3390/molecules25030617

**Published:** 2020-01-31

**Authors:** Wim Wouter Oomen, Paloma Begines, Natali Rianika Mustafa, Erica G. Wilson, Robert Verpoorte, Young Hae Choi

**Affiliations:** 1 Natural Products Laboratory, Institute of Biology, Leiden University, Sylviusweg 72, 2333 BE Leiden, The Netherlands; wim.oomen@hotmail.com (W.W.O.); pbegines@us.es (P.B.); mustafa@chem.leidenuniv.nl (N.R.M.); erica.g.wilson@gmail.com (E.G.W.); verpoort@chem.leidenuniv.nl (R.V.); 2 College of Pharmacy, Kyung Hee University, Seoul 02447, Korea

**Keywords:** NADES, extraction, ionic liquid, lipophilic compounds, HPTLC, HPLC, phenolic compounds

## Abstract

Natural deep eutectic solvents (NADES) are a type of ionic liquid (IL) or deep eutectic solvent (DES), the ingredients of which are exclusively natural products (non-toxic and environmentally friendly). Here, we explore the potential of NADES as an alternative to conventional organic solvents (e.g., aqueous methanol or ethanol) for the extraction of flavonoids from *Scutellaria baicalensis* stem bark to investigate their extractability depending on structural variation. Four NADES, each containing citric acid in combination with β-alanine, glucose, xylitol, or proline (at a molar ratio of 1:1), and a variable amount of water, were used to extract the flavonoid aglycones: baicalein (**1**), scutellarein (**3**), wogonin (**5**), and oroxylin A (**7**), and their glycosides, baicalin (**2**), scutellarin (**4**), wogonoside (**6**) and oroxyloside (**8**) from the powdered bark of *S. baicalensis*. The chemical profile and yield of the extracts were determined using HPTLC and HPLC. The extractability of individual flavonoids was found to be influenced by the concentration of water (20–60%, w/w) in the NADES. Among the tested flavonoids, the extraction yield of baicalein (**1**), scutellarein (**3**), wogonin (**5**), oroxylin A (**7**) with NADES was 2 to 6 times that of aqueous methanol. However, the amount of their corresponding glycosides (baicalin (**2**), wogonoside (**6**) and oroxyloside (**8**)) extracted with NADES was only 1.5–1.8 times higher than with aqueous methanol. Interestingly, the more hydrophilic glycosides were less extracted than their corresponding aglycones despite the high hydrophilicity of the NADES. These results prove that NADES may be used for extraction of compounds with a wide range of hydrophilicity.

## 1. Introduction

Organic solvents are considered to be irreplaceable in many industries, being used in many processes e.g., for the production of medicines, extraction of colorants and dyes, purification of compounds from diverse matrixes and numerous other applications. Unfortunately, despite their toxicity and the environmental hazard they pose, organic solvents are still used in great amounts, with high costs involved in their waste disposal. It has thus been some time now that the industry has been attempting to replace them with greener technology. There are four major strategies to avoid the use of conventional harmful or toxic organic solvents. These are: 1) the use of heterogeneous catalysts, 2) water, 3) supercritical fluids or 4) ionic liquids (ILs) [1], as well as 5) a recently emerging field, the application of nanoparticles to the extraction of various samples, which has been reviewed for environmental applications [2], food toxins [3] and biological fluids [4]. With the exception of the use of catalysts, all the alternatives are associated with the use of non- or at least less toxic solvents. However, these main strategies have not had the expected success and the search for a really ‘green’ substitute for the currently used solvents is still needed. Thus, over the last decade, in the field of green technology, ILs and their related technologies have been actively applied across a wide range of fields [5].

Ionic liquids are ionic mixtures, the state of which changes from solid to liquid around or below 100 °C [1]. One of the main characteristics of ILs is that they have no detectable vapor pressure, and consequently do not evaporate in ambient conditions. ILs are characterized by the existence of ionic bonds between their components, which require at least one cation and one anion. However, the application of ILs in the food industry is limited because many of them are based on imidazolium and pyridinium species and are therefore toxic. A subclass of solvents that have similar eutectic properties are deep eutectic solvents (DES), first described in 2001 by Abbot and his colleagues [6]. A DES is a liquid that is formed when at least two certain solid compounds are mixed in conditions that lower their melting points to a point low enough to form a eutectic mixture [7,8]. In 2011, Choi and colleagues proposed that there might be DES-like media in nature playing many biological roles and hypothesized that this new kind of DES, they named natural deep eutectic solvents (NADES) might be present in living organisms. Natural deep eutectic solvents are natural solvents based on the principle of ILs and DES, but primarily composed of natural compounds. They thus constitute a very promising option for green chemistry and are candidates to replace the toxic organic solvents [9].

Among many NADES applications, the most extensively studied refer to the extraction of natural products. In particular, very diverse natural phenolic compounds have been extracted with NADES, very successfully in most cases. For example, extraction of anthocyanins from grape skins with a NADES consisting of citric acid-maltose (4:1; mole/mole) showed a yield that practically doubled that obtained with 80% methanol [10]. Other successful applications include the extraction of trans-resveratrol from roots of *Polygoni cuspidati* [11], *C*-glucosyl quinochalcones from flowers of *Carthamus tinctorius* [12], anthocyanins from grape pomace [13] and phenolic antioxidants from Greek medicinal plants [14]. Alongside the extraction yields, Dai and colleagues also found that NADES could not only increase the extraction yield but also the stability target compounds [15]. Moreover, by tailoring the water concentrations in NADES, Dai and colleagues laid the basis for a manageable way of applying NADES in food processing, enzymatic reactions and pharmaceutical and cosmetic applications [16]. Recent applications of NADES to diverse fields have been reviewed by Vanda and colleagues [17].

In this study, the feasibility of using NADES to extract phenolics, particularly flavonoids, was further investigated. Roots of *Scutellaria baicalensis* (Labiatae) were selected as model material. This plant is one of the most popular herbal drugs in traditional Chinese medicine (TCM) and is often used for the treatment of cardiovascular disease and bleeding disorders as well as for some infectious diseases [18,19]. The main active compounds of the plant have been reported to be flavonoids and so far, over 40 analogues have been identified in this plant material [20]. 

Although NADES are very promising solvents, some of their inherent features such as high viscosity and low vapor pressure make their evaluation difficult. Thus, a sample preparation procedure and analytical method that can circumvent these difficulties is needed to analyze the extracts. Recently, a method based on high performance thin layer chromatography (HPTLC) coupled to multivariate data analysis (MVDA) was developed for the analysis of NADES plant extracts [21]. Previously, for the analysis of plant extracts, HPTLC-based methods were shown to be easily applied to both targeted and non-targeted studies aimed at obtaining their chemical profiles [22,23]. For NADES extracts, the HPTLC-based method provided a quick and reliable method to perform the ‘fingerprint’ analyses of the extracts, as well as a broad coverage of metabolites [21]. 

In this study, roots of *S. baicalensis* were extracted with several NADES, followed by chemical assessment by HPTLC in an untargeted manner. In addition to HPTLC analysis, a target analysis of the major flavonoids was performed by HPLC-UV. 

The results of this study allowed us to evaluate both the extraction efficiency of the selected NADES and their extraction profiles. Apart from the extraction capacity of the different NADES, the effect of water addition in NADES was also evaluated. 

## 2. Results and Discussion

**Figure 1:**
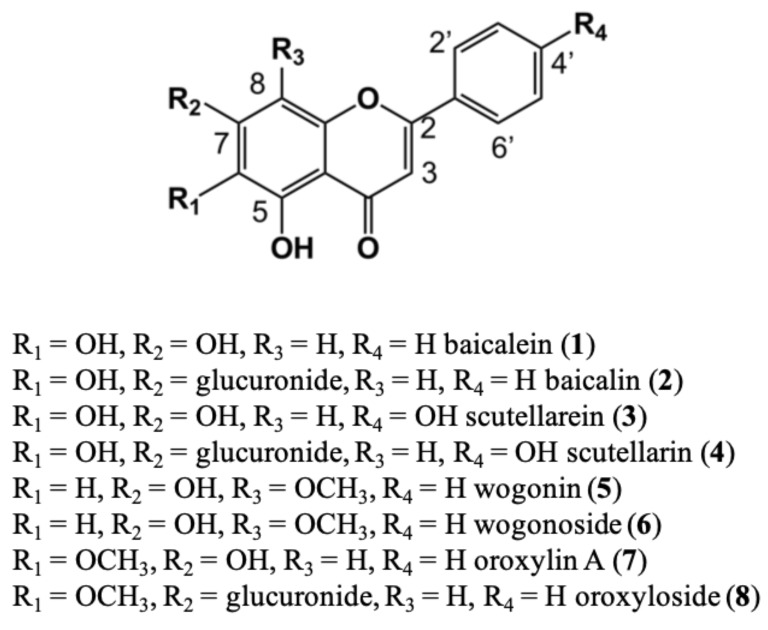
Chemical structures of the flavonoids of *Scutellaria baicalensis* employed in the study.

In this study, four different NADES and their capability to extract flavonoids from *S. baicalenisis* was tested. Following previous work by González and colleagues [24], all four selected NADES were based on citric acid because they were non-toxic and had potential to extract phenolics. The selected NADES were 1:1 molar ratio mixtures of β-alanine–citric acid (N1), glucose–citric acid (N2), xylitol–citric acid (N3) and proline–citric acid (N4). All of the components were apt for consumption, to prove that NADES-based extracts can be used directly for food or pharmaceutical applications. Among the many flavonoids present in *Scutellaria baicalensis,* eight of the major components were chosen: baicalein (**1**), scutellarein (**3**), wogonin (**5**), oroxylin A (**7**) and their glycosides, baicalin (**2**), scutellarin (**4**), wogonoside (**6**) and oroxyloside (**8**) [25] (Figure 1).

The inherent viscosity of most NADES requires a relatively long extraction time and can lead to some difficulties in post-extraction procedures, e.g., residual solvents in the extracts interfering with the analysis. There are two ways to solve this problem: to increase the working temperature or add a certain percentage of water. It has been observed that even adding very small amounts of water can result in a dramatic decrease in the viscosity of most NADES. Thus, 10% of water was added to the NADES as suggested by Dai and colleagues [16]. This proved to successfully lower the viscosity and partly solve the problems during extraction, but the recovery of metabolites from the NADES extract using a solid phase extraction (SPE) column still proved to be rather difficult even with this relatively lower viscosity.

The comparison of extraction patterns was firstly performed using HPTLC whereas HPLC was applied for quantitative analysis by targeting the selected flavonoids. The advantage of using HPTLC is that it allows the simultaneous analysis of multiple samples on one plate, facilitating their comparison. A disadvantage is that given the difference in hydrophilicity of the targeted flavonoid aglycones and their glycosides, it is no less hard to obtain an acceptable resolution on the same plate with the same chromatographic system. Thus, it is not possible to capture all flavonoids in one HPTLC image. To solve the problem, we ran the HPTLC experiment with two different mobile phases, managing to identify extraction ‘fingerprints’ which were then categorized based on principle component analysis (PCA). PCA is necessary to analyze multiple variables in the HPTLC image (e.g., Rf-value, color and intensity) in order to reveal the difference among the observed groups (different extraction profiles). It was not possible to separate all flavones and their glycosides with the HPTLC system, so that quantitative analysis of the content of each of these compounds was done using HPLC. The HPLC results confirmed the presence of seven of the targeted flavonoids (baicalein (**1**), scutellarein (**3**), wogonin (**5**), oroxylin A (**7**) and the glycosides baicalin (**2**), wogonoside (**6**) and oroxyloside (**8**)), while the glycoside scutellarin (**4**) was undetectable in all of the extracts. This differed from the results obtained by Liu and colleagues, who reported the presence of scutellarin in *Scutellaria baicalensis* [25].

### 2.1. Comparison of Extraction Profiles and Yields between Different NADES and Organic Solvents

**Figure 2:**
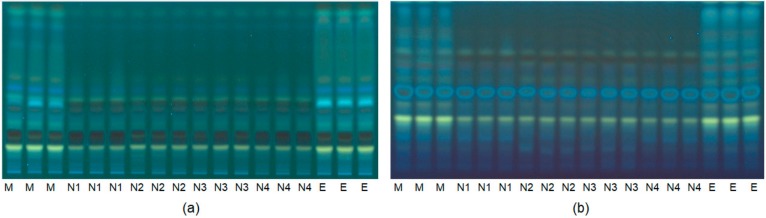
High-performance thin-layer chromatograms (HPTLC) of *Scutellaria baicalensis* bark extracted with 80% methanol in water, 70% ethanol in water and natural deep eutectic solvents (NADES) visualized at 366 nm after derivatization with natural products reagent (NPR) with two different mobile phase conditions. (**a**) Chromatogram obtained with the mobile phase: ethyl acetate, methanol, formic acid, water (EMFW, 20:2.7:0.5:2, ratio *v/v/v/v*); (**b**) Chromatogram obtained with the mobile phase: ethyl acetate, formic acid, acetic acid and water (EFAW, 100:11:11:27, *v/v/v/v*). M: 80% methanol in water extract (*v/v*), E: 70% ethanol in water (v/v), N1–N4: NADES with the addition of 10% water (*w/w*). N1: citric acid–β-alanine (1:1, mole/mole). N2: citric acid–glucose (1:1, mole/mole). N3: citric acid–xylitol (1:1, mole/mole). N4: citric acid–proline (1:1, mole/mole).

**Figure 3:**
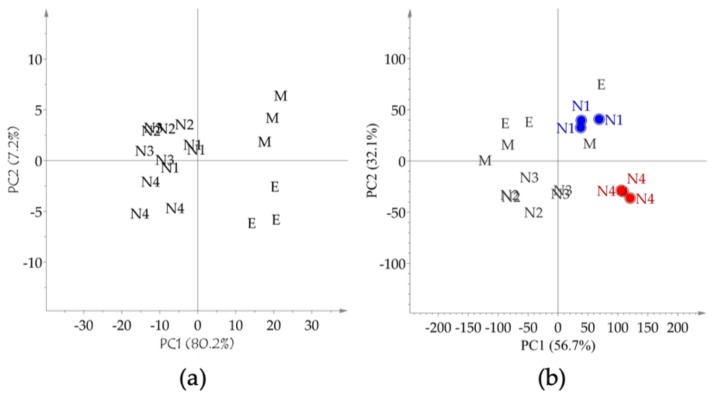
Score plot of principle component analysis of the HPTLC (**a**) and HPLC (**b**) data obtained from the extracts of 80% aqueous methanol, 70% aqueous ethanol, and natural deep eutectic solvent (NADES) extracts of *Scutellaria baicalensis* bark. M: 80% methanol in water extract (*v/v*), E: 70% ethanol in water (*v/v*), N1–N4: NADES with the addition of 10% water (*w/w*). N1: citric acid–β-alanine (1:1, mole/mole). N2: citric acid–glucose (1:1, mole/mole). N3: citric acid–xylitol (1:1, mole/mole). N4: citric acid–proline (1:1, mole/mole).

In this work, four selected edible NADES were tested for their flavonoid extraction capability. Following the determination of the overall extraction yield of the targeted flavonoids, the two most efficient NADES were selected for further studies. The organic solvents used as controls were 80% (*v/v*) aqueous methanol and 70% (*v/v*) aqueous ethanol. Firstly, the HPTLC fingerprint extraction pattern was developed as shown in Figure 2. At first sight, the control aqueous ethanol and methanol profiles were more intense in comparison to the NADES profiles. However, a closer inspection of the profiles, revealed differences in the extraction profiles of the NADES and the control extracts with an increase in certain flavonoids. The HPTLC bands were further analyzed by PCA after data processing and the score plot revealed a similarity in the extraction capacity of the two control solvents and a similarity amongst the different NADES (Figure 3a). Interestingly, the NADES, N4 with citric acid–proline (1:1, mole/mole), was found to have a slightly different profile to those of the other NADES, N1 (citric acid–β-alanine, 1:1, mole/mole), N2 (citric acid–glucose, 1:1, mole/mole), and N3 (citric acid–xylitol, 1:1, mole/mole). Further observations on the similarities and differences between the extracts, the HPLC data targeted for the selected flavonoids was analyzed by PCA, for which the peak intensities of eight flavonoids were used as variables (Figure 3b). The score plot of PCA showed that N1 and N4 had clearly distinct extraction profiles.

The HPLC analyses were used to compare the extraction profile of each NADES and their quantitative composition in targeted flavonoids. The yield of total flavonoids obtained with each NADES (N1 to N4), based on the peak integrals of flavonoids, were 80%–170% that of aqueous methanol or ethanol extracts. A higher yield of the targeted flavonoids was also observed in N1 and N4 extracts so these NADES were selected for further studies involving the effect of water addition.

### 2.2. Effect of Different Water Concentrations in NADES on Flavonoid Extraction 

It has been reported that water content is one of the factors that most affects NADES extraction capacity [16]. Thus, in this second part, the influence of different water concentrations in N1 and N4, which showed promising results, on their flavonoid extraction capacity was explored. The correlations between solubility or extractability, and water content are not necessarily linear and, in many cases, the optimum solubility or extractability is found only at a specific water content in NADES [16]. 

**Figure 4:**
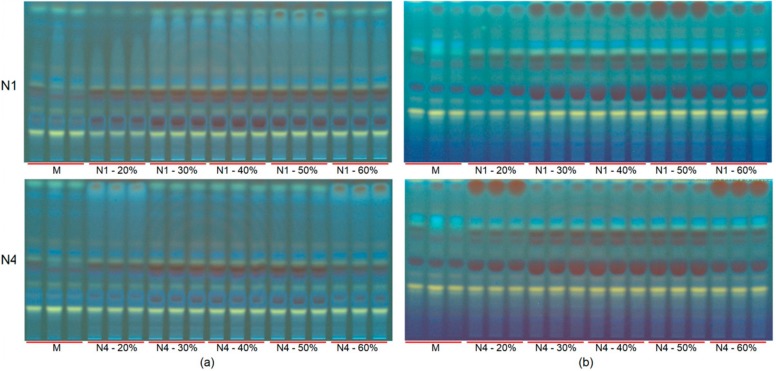
High-performance thin-layer chromatograms (HPTLC) of extracts of *S. baicalensis* bark obtained with 80% methanol in water and two NADES with different concentrations of water visualized at 366 nm after derivatization with natural products reagent (NPR) with two different mobile phase conditions. (**a**) Chromatogram obtained with mobile phase: ethyl acetate, methanol, formic acid, water (EMFW, 20:2.7:0.5:2, ratio *v/v/v/v*); (**b**) Chromatogram obtained with mobile phase: ethyl acetate, formic acid, acetic acid and water (EFAW, 100:11:11:27, *v/v/v/v*). M: 80% aqueous methanol (*v/v*), N1-20: citric acid–β-alanine (1:1, mole/mole) with the addition of 20% (*w/w*), N1-30: citric acid–β-alanine (1:1, mole/mole) with the addition of 30% (*w/w*), N1-40: citric acid–β-alanine (1:1, mole/mole) with the addition of 40% (*w/w*), N1-50: citric acid–β-alanine (1:1, mole/mole) with the addition of 50% (*w/w*), N1-60: citric acid–β-alanine (1:1, mole/mole) with the addition of 60% (*w/w*), N4-20: proline–citric acid (1:1, mole/mole) with the addition of 20% (*w/w*), N4-30: proline–citric acid (1:1, mole/mole) with the addition of 30% (*w/w*), N4-40: proline–citric acid (1:1, mole/mole) with the addition of 40% (*w/w*), N4-50: proline–citric acid (1:1, mole/mole) with the addition of 50% (*w/w*), N4-60: proline–citric acid (1:1, mole/mole) with the addition of 60% (*w/w*).

**Figure 5:**
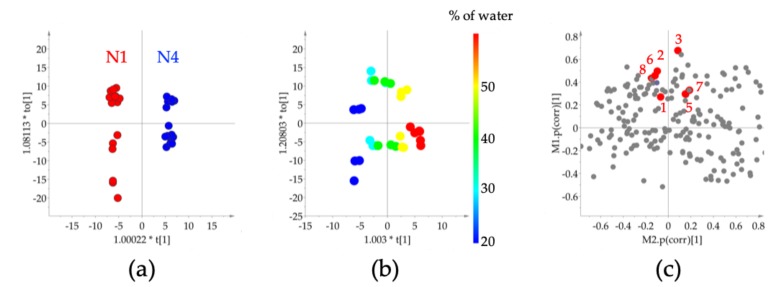
Orthogonal partial least square modeling of HPTLC and HPLC data of *Scutellaria baicalensis* roots with the kind of NADES (N1 and N4) and the percentage of water added to NADES. (**a**) Score plot of OPLS-DA for the effect of the kind of NADES (N1 and N4), (**b**) Score plot of OPLS for the effect of the percentage of added water, (**c**) SUS plot to combine two models (the kinds of NADES and the percentage of water). N1: citric acid–β-alanine (1:1, mole/mole). N4: citric acid–proline (1:1, mole/mole). Compounds in SUS plot (c): baicalein (**1**), scutellarein (**3**), wogonin (**5**), oroxylin A (**7**) and their glycosides, baicalin (**2**), wogonoside (**6**) and oroxyloside (**8**).

This test was carried out by extracting the sample with the two best performing NADES, N1 and N4, to which increasing amounts of water (20%–60%, *w/w*) were added. The HPTLC fingerprints clearly revealed the extent of the influence of the water content on the yield of the individual compounds (Figure 4). A possible explanation for this is the increased hydrophilicity provided by the water content in the NADES that favors the extractability of the flavonoids. To investigate the effect of water on the extraction profile, orthogonal partial least square (OPLS) modeling was applied to the data of HPTLC and HPLC, for which the percentage of added water or the kind of NADES (N1 and N4) was used as the Y-variable and the combined HPLC and HPTLC data were treated as single X-data. As shown in Figure 5A,B, the extraction profile was greatly influenced by the NADES character (N1 and N4) and water content. The two OPLS models, each of which is for water effect and the characters of NADES, respectively were combined and the SUS plot was employed to interpret the individual effect on each flavonoid (Figure 5C, *X*-axis: NADES characters, *Y*-axis: water content). Most glycosides were influenced more by water content than aglycones, except for scutellarein (**3**). Interestingly, each NADES showed different effects; N1 was found to extract more glycosides such as baicalin (**2**), wogonoside (**6**) and oroxyloside (**8**), but N4 showed better extraction yields for aglycones (**3**), wogonin (**5**) and oroxylin A (**7**).

The HPLC analyses of the extracts showed that, as expected, the flavonoid aglycones and their corresponding glycosides were not best extracted at the same water concentration. Unexpectedly, the higher percentage of water did not increase the yield of the more hydrophilic glycosides. There might be several reasons for this phenomenon. The addition of water does not greatly change hydrophilicity of NADES because the components of NADES are already hydrophilic enough and thus not affected by the addition of water. Secondly, the extraction or solubilization of flavonoids does not follow the increase of hydrophilicity of the solvents in a proportional manner, that is, an optimum hydrophilicity might be attained with a specific amount of water Actually, some phenolics are best extracted with specific water contents [16].

All of the tested NADES were able to extract flavonoids, some being more efficient than conventional organic extraction (Table 1). The maximum increase in the yield of baicalein (**1**), scutellarein (**3**), wogonin (**5**) and oroxylin A (**7**) was approximately 3–5 times that of 80% methanol extraction. On the other hand, the corresponding glycosides of these flavonoids showed a lower increase in their extraction yields with NADES of approximately 1.5-fold when compared with the organic solvent extraction.

In this study, the extraction of phenolic compounds from *S. baicalensis* bark with citric acid-based NADES was explored with the expectation of providing further evidence for the development and establishment of NADES as universal green solvents for natural products. Proving that NADES are a viable alternative to conventional organic solvents (e.g., aqueous ethanol and methanol) for the extraction of flavonoids was a step in this direction. The highest increase in total flavonoids yield was obtained with N1: citric acid–β-alanine (1:1, mole/mole) with 50% water (*w/w*). N4: citric acid–proline (1:1, mole/mole) with 40% water. It is noteworthy that both N1 and N4 contained an amino acid as one of its ingredients, which might be advantageous as food ingredients. Among the tested flavonoids, the extractability of wogonin with N4 with 60% water (*w/w*) was particularly notable, with a 6.32-fold increase compared to aqueous methanol.

We report the use of a simple method that allows the simultaneous analysis of multiple extracts obtained with different NADES using HPTLC and HPLC. The viscosity of the NADES is still a major obstacle in the recovery of metabolites from the extracts and efforts to provide a technique or process to improve this should be encouraged. The extended range of hydrophilicity/lipophilicity of the extracted compounds achieved in this case with the NADES (with contents between 20 and up to 60% of water) is also very promising.

## 3. Materials and Methods 

### 3.1. Chemicals and Materials 

All of the chemicals for the preparation of NADES, including β-alanine, citric acid, glucose, xylitol and proline, were analytical grade and purchased from Sigma Aldrich (St. Louis, MO, USA). Analytical standards of scutellarein, scutellarin, baicalein, baicalin, wogonin, wogonoside, oroxylin A and oroxyloside (> 95% purity) were purchased from Biopurify (Chengdu, China). HPLC grade acetonitrile, water and formic acid were purchased from Honeywell Riedel-de Haën (Seelze, Germany). Dried roots of *Scutellaria baicalensis* Geroge were collected in Hubei (Wuhan, China) (voucher Specimen No. NPL-R-KDSB-2051210, NPL), purchased from Kyung-Dong market (Seoul, Republic of Korea), and identified by Dr. Y. H. Choi. 

### 3.2. Preparation of NADES

Citric acid-based natural deep eutectic solvents employed in this study were made by mixing the ingredients of each NADES at a fixed molar ratio (1:1). These were β-alanine–citric acid (N1), glucose–citric acid (N2), xylitol–citric acid (N3) and proline–citric acid (N4). The ingredients of NADES were dissolved with a minimum amount of water, followed by heating while stirring at 50 °C. The obtained liquids were dried using a vacuum rotary evaporator at 50 °C for 4–6 h (Büchi, Hendrik-Ido-Ambacht, The Netherlands) and residual water was removed by 24 h of freeze-drying. For the first screening experiment, for the selection of the optimum NADES, 10% (w/w) of water was added to each NADES, followed by stirring at 40 °C until a homogenous liquid was formed [9,26].

For the optimization of water content in NADES, N1 (β-alanine–citric acid: 1:1) and N4 (proline–citric acid: 1:1) were selected and 20%, 30%, 40%, 50% and 60% (*w/w*) of water was added to each of them, followed by stirring and heating at 50 °C. 

### 3.3. Extraction of Plant Material

Mixing of plant material and NADES was performed in a 2 mL-microtube with 100 mg dry weight (DW) of powdered plant material and 2 mL of NADES. For the control experiments, 70% (*v/v*) ethanol or 80% (*v/v*) methanol was used instead of the NADES. The mixture was vortexed for 1 min, placed into a water bath (at 40 °C for 1 h), ultrasonicated for 30 min at room temperature, and centrifuged at 13,000 rpm for 20 min. From the extracts, 1.0 mL of supernatant was removed and mixed with 1.0 mL of water and homogenized/vortexed for 1 min. This mixture was submitted to a sample preparation/clean-up process using solid phase extraction (SPE). An aliquot of 1.0 mL of the control extracts obtained with aqueous ethanol and methanol was dried in a vacuum concentrator and redissolved in 2 mL of water before applying it on the SPE column. All extracts were prepared by triplicate.

### 3.4. Recovery of Compounds from NADES with Solid Phase Extraction (Sample Clean Up)

Solid phase extraction (SPE) was carried out in 5 mL Oasis Hydrophilic-Lipophilic-Balance (HLB) cartridges (Waters Corporation, Milford, MA, USA). The HLB cartridges were placed in a vacuum and equilibrated with 5.0 mL methanol followed by 5.0 mL of water. After loading the 2 mL of the sample, the cartridges were flushed with 19 mL of water and then eluted with 6.0 mL of methanol. The methanol fractions were dried in a rotary evaporator and redissolved with 2.0 mL of methanol. Of these fractions, 1.0 mL was used for the HPTLC analysis and the other 1 mL for HPLC analysis.

### 3.5. HPTLC Analysis

A CAMAG high performance thin layer chromatography (HPTLC) system equipped with an automatic TLC sampler (version 4), a derivatizer device (version 1.0 AT), a TLC plate heater (version III) and a TLC visualizer (CAMAG, Muttenz, Switzerland) was used for analysis. The separation was carried out on 20 × 10 cm F254 silica gel 60HPTLC plates (Merck, Darmstadt, Germany). Five μL of the solution were spotted in 6 mm bands with a distance of 10 mm from the bottom, 20 mm from the left and right edge, the distance between bands was 8.8 mm, allowing 18 samples per plate. Two different mobile phases were used for the analysis: ethyl acetate, formic acid, acetic acid and water (100:11:11:27, *v/v/v/v*) and ethyl acetate, methanol, formic acid and water (20:2.7:0.5:2, *v/v/v/v*) with a chamber saturation time of 20 min. The developed plates were dried with an air-stream at room temperature and scanned with the CAMAG TLC visualizer at 254, 366 nm and white light. The plates were also derivatized with 2 mL of a solution of 1g of 2-aminoethyl diphenylborinate in 100 mL of methanol (NP reagent) and after 5 min of reaction viewed at 366 nm with a CAMAG TLC visualizer.

### 3.6. HPLC Analysis 

An Agilent Technologies Series 1200 chromatographic system (Santa Clara, CA, USA) equipped with a photodiode array detector (DAD) was used for HPLC analysis. Analysis was performed on a Luna C18 (2) (100 mm × 4.6 mm, 5 µm) (Phenomenex, Utrecht, The Netherlands) column and samples were eluted with a gradient of 0.1% formic acid in water (solvent A) and 0.1% formic acid in acetonitrile (solvent B) as follows: 15% B (0–13 min), 15–75% B (13–14 min), 75–15% B (14–14.10 min), 15% (14.10–15.10 min) at a flow rate of 1 mL/min. The injection volume for reference compounds and samples was 10 µL. The chromatogram was recorded at 280 nm and 340 nm for quantitation. All HPLC analyses were conducted in triplicate.

For water content optimization analyses, a different column, Phenomenex Luna C18(2) (150 mm × 4.6 mm, 5 µm), was used with the same solvents and gradient program as mentioned above. 

### 3.7. Data Processing of HPTLC and HPLC Data and Multivariate Data Analysis

The HPTLC chromatogram scans were processed using the online rTLC software [27]. The generated numerical data of the gray-scale was used in the Rf range between 0.03 and 0.958. The HPTLC data of chromatograms obtained with both mobile phases were analyzed with the SIMCA-P software (version 15, Umetrics, Umeå, Sweden) for principal component analysis (PCA) using Pareto or UV scaling method.

## Figures and Tables

**Table 1:** Yield of individual flavonoids extracted from *Scutellaria baicalensis* root in NADES solvents N1 (citric acid—β-alanine, 1:1) and N4 (citric acid—proline. 1:1) with different water concentrations. Solvents in µg/mg plant extract ± SD based on triplicates.

Solvent *	baicalein (1)	baicalin (2)	scutellarein (3)	wogonin (5)	wogonoside (6)	oroxylin A (7)	oroxyloside (8)
M	1.4 ± 0.2	27.8 ± 1.8	6.4 ± 0.7	14.1 ± 1.2	14.9 ± 0.4	1.1 ± 0.2	2.0 ± 0.3
E	1.3 ± 0.4	24.9 ± 4.8	5.1 ± 1.5	11.3 ± 4.0	21.9 ± 7.8	1.3 ± 0.5	2.4 ± 0.8
N1-20%	1.4 ± 0.4	26.2 ± 4.8	3.8 ± 0.2	8.8 ± 0.7	24.1 ± 2.2	1.0 ± 0.3	2.0 ± 0.3
N1-30%	1.8 ± 0.3	25.3 ± 2.1	6.4 ± 0.4	17.1 ± 0.8	51.9 ± 0.4	2.4 ± 0.2	4.8 ± 0.3
N1-40%	2.7 ± 0.8	39.4 ± 9.5	7.5 ± 0.9	18.6 ± 1.3	59.4 ± 4.5	2.9 ± 0.4	5.4 ± 0.7
N1-50%	3.0 ± 0.6	38.1 ± 4.6	6.8 ± 0.4	16.7 ± 0.4	73.3 ± 0.9	4.7 ± 1.0	8.6 ± 0.5
N1-60%	2.6 ± 0.3	37.9 ± 1.8	6.9 ± 0.3	17.1 ± 0.8	39.6 ± 1.5	2.2 ± 0.3	3.9 ± 0.3
N4-20%	2.3 ± 0.3	28.8 ± 1.9	3.8 ± 0.4	8.2 ± 0.7	73.9 ± 1.7	6.2 ± 0.5	9.3 ± 0.6
N4-30%	2.0 ± 0.5	31.8 ± 5.5	6.6 ± 0.5	17.1 ± 0.6	21.1 ± 0.8	1.4 ± 0.2	2.6 ± 0.3
N4-40%	2.4 ± 0.6	36.4 ± 7.5	7.3 ± 1.0	18.6 ± 2.3	25.5 ± 5.8	1.7 ± 0.5	3.2 ± 0.8
N4-50%	2.0 ± 0.3	27.0 ± 2.5	7.1 ± 0.3	19.4 ± 0.4	30.8 ± 3.0	2.0 ± 0.3	3.5 ± 0.3
N4-60%	3.2 ± 0.2	32.0 ± 1.3	4.6 ± 0.4	10.9 ± 0.7	82.4 ± 2.1	7.0 ± 0.7	12.0 ± 1.1

* M: 80% methanol, E: 70% ethanol, % next to N1 or N4 is the percentage of added water to NADES (*w/w*).
